# Beyond livestock: the genomic evolution and expansion of MRSA CC398 as a healthcare- and community-associated pathogen (2017–2024)

**DOI:** 10.1099/mgen.0.001741

**Published:** 2026-06-02

**Authors:** Jan Tkadlec, Adela Rozsypalkova, Sylvia Polivkova, Pavel Drevinek, Jairo Gooskens, Marcela Krutova

**Affiliations:** 1 Department of Medical Microbiology, Second Faculty of Medicine, Charles University and University Hospital Motol and Homolka, Prague, Czech Republic; 2 Department of Infectious Diseases and Tropical Medicine, Second Faculty of Medicine, Charles University and University Hospital Motol and Homolka, Prague, Czech Republic; 3 Center of Infectious Diseases (LUCID), Leiden University Medical Center, Leiden, Netherlands

**Keywords:** CA-MRSA, CC398, LA-MRSA, One Health, ST1232, ST398

## Abstract

This study employed whole-genome sequencing to elucidate the genomic diversity and population structure of *Staphylococcus aureus* clonal complex 398 (CC398) in the Czech Republic over a 7-year period (2017–2024). Among 1,129 MRSA isolates from human patients, 59 CC398 isolates were identified and characterized via core-genome SNP and whole-genome MLST-based phylogenetic reconstruction. The results revealed two phylogenetically and clinically divergent lineages. The ST1232 lineage (*n*=27), a human-adapted community-associated (CA) MRSA clone harbouring Panton–Valentine leucocidin and immune evasion cluster (IEC) type B, showed a sharp increase in prevalence after 2022. Phylogenetic analysis suggested multiple independent introductions linked to international travel, primarily to South-East Asia, followed by local expansion within households and among vulnerable populations. In contrast, the ST398 lineage (*n*=32) comprised diverse sublineages, including a swine-associated clone (SCC*mec* Vc) and an equine-associated sublineage (C6-EP5-Leq; SCC*mec* IVa, IEC type E). ST398 was primarily associated with healthcare-related colonization and long-term persistence in patients with chronic conditions. Our data demonstrate the genomic diversification of CC398 into specialized niches, with the expansion of the virulent ST1232 lineage mirroring the early evolutionary trajectory of successful CA-MRSA clones such as USA300. This study highlights the increasing complexity of CC398 epidemiology, characterized by the simultaneous expansion of livestock-derived sublineages and human-adapted clades, such as ST1232 and underscores the necessity of continuous genomic surveillance within a 'One Health' framework.

**Impact Statement**

This study provides a high-resolution, 7-year genomic and clinical characterization of *Staphylococcus aureus* clonal complex 398 in Central Europe, documenting its critical transition from a predominantly livestock-associated threat into a multifaceted population comprising both zoonotic and established human-adapted lineages. Using core-genome SNP and whole-genome MLST analyses, we demonstrate the emergence and steady expansion of the virulent, human-adapted ST1232 lineage, which is now established in the community and increasingly linked to vulnerable populations. Our findings reveal that ST1232 mirrors the early transmission dynamics of successful clones like USA300, posing a significant risk for a major community-associated epidemic. Furthermore, we identify an equine-associated ST398 sublineage circulating in healthcare environments, highlighting how clinical niches with high antimicrobial pressure facilitate cross-species transmission and persistence. This work underscores the necessity of genomic surveillance at the human–animal–environment interface to monitor the evolving 'One Health' threat of increasingly virulent MRSA sublineages.

## Data Summary

The data supporting the findings of this study are available within the article and its supplementary data. Raw read sequences of all isolates were submitted to the NCBI Sequence Read Archive under BioProject accession number PRJNA1367295 and could be found using the accession numbers provided in Table S1, available in the online Supplementary Material.

## Introduction

Among *Staphylococcus aureus* lineages, clonal complex 398 (CC398) is particularly notable for its position at the human-animal interface. Following a split from an ancestral human-associated methicillin-susceptible *S. aureus* population dated between 1957 and 1970 [1,2], livestock-associated methicillin-resistant *S. aureus* (LA-MRSA) emerged in the early 2000s in Europe [3,4], subsequently diversifying into several animal-adapted sublineages [5,6]. Over the last decade, human-origin MRSA ST1232, belonging to CC398, initially described in the Asia-Pacific region [7,8], emerged across Europe, linked to outbreaks in Denmark [9] and sporadic cases in the Netherlands [10], Germany [11] and Hungary [12]. While recent reports have identified Panton–Valentine leucocidin (PVL)-positive ST1232 in Czech hospitals [13], its long-term clinical impact and transmission dynamics in Central Europe remain poorly characterized due to a lack of high-resolution longitudinal data.

This study provides a 7-year (2017–2024) genomic and clinical characterization of CC398 MRSA in the Czech Republic. Our objectives were to (i) investigate the evolving molecular epidemiology and clinical burden in human patients, (ii) define the genomic landscape of virulence and resistance determinants and (iii) identify critical reservoirs and transmission pathways at the human–animal–environment interface.

## Methods

### Isolate collection and antimicrobial susceptibility

The study included 59 non-repetitive isolates from 56 patients belonging to CC398, collected from 5 Czech hospitals participating in our previous study in 2017 (*n*=11) [14] and at Motol University Hospital (*n*=48) between 2018 and 2024 (including two methicillin-susceptible PVL-positive CC398 isolates). Isolates from Motol University Hospital were collected as part of long-term MRSA surveillance. One isolate per patient per year was included and subjected to *spa* typing [15]. Association with CC398 was derived from *spa* typing results. An isolate was considered non-repetitive even if obtained from a previously sampled patient, provided it was collected in a different calendar year [16], under a distinct clinical indication, and from a different anatomical site. Antimicrobial susceptibility to oxacillin, cefoxitin, erythromycin, clindamycin, gentamicin, tetracycline, rifampicin, ofloxacin, trimethoprim-sulfamethoxazole, linezolid, tigecycline and ceftaroline was tested via the disc diffusion method, including D-tests for inducible clindamycin resistance, with the breakpoints recommended by EUCAST [17].

For each patient, the following clinical and epidemiological metadata were retrospectively collected from electronic medical records: age, sex, hospital unit type, clinical presentation, underlying conditions, healthcare contact within the preceding 12 months, recent travel history, household MRSA contacts and contact with socially disadvantaged groups. Metadata are provided in Table S1.

### Whole-genome sequencing

Genomic DNA from an individual bacterial colony was extracted using MasterPure Complete DNA and RNA Purification Kit (Biosearch Technologies, Hoddesdon, UK). All 59 isolates were sequenced using the Illumina NextSeq 2000 platform. Additionally, 16 representative isolates selected to span all major phylogenetic clusters were sequenced using the Oxford Nanopore MinION platform to generate hybrid assemblies for complete genome characterization. Short reads were assembled with SPAdes v3.15.5 [18], while hybrid assemblies were generated using Flye (v2.9.5), Medaka (v1.9.1) and Polypolish (v0.5.0) [19,20]. Genomes were annotated using Bakta (v1.9.4) [21]. Assembled sequences were screened for antimicrobial resistance genes [22], virulence determinants [23,24], mobile genetic elements (MGEs) [25] and SCC*mec* types [26] using Center for Genomic Epidemiology tools. Prophages were identified using PHASTEST (v3.0) [27]. Genetic relatedness was assessed via whole-genome MLST (wgMLST) based on 3,897 loci (BioNumerics v8.1). The minimum spanning tree (MST) was constructed using character data using a categorical coefficient, where each locus was treated as a categorical variable. Missing loci (assigned as zero values) were not ignored (setting 'ignore zero values: No') and were instead treated as a distinct character state. This approach ensured that variation in gene presence/absence, in addition to allelic polymorphism, is reflected in the final tree topology. Phylogenetic reconstruction was based on *de novo* assembled genomes; a core-genome alignment was generated using Roary (v3.13.0) [28], followed by the identification and removal of recombinant regions with Gubbins (v3.2.1); a maximum likelihood (ML) phylogeny was inferred using RAxML (v8.2.12) as implemented in Gubbins [29]. Phylogenetic trees were visualized in iTOL (Interactive Tree of Life, v7.2.1) [30]. A threshold of ≤24 allelic differences in wgMLST was used to assume close genetic relatedness [31]. Detailed parameters and specific settings for all bioinformatic tools used in this study are provided in Table S2.

For global phylogenetic reconstruction, 58 publicly available genomes were included in the core-genome SNP (cgSNP) analysis. These comprised 26 reference strains representing established CC398 phylogenetic lineages as defined by Fernandez *et al*. [5] and Price *et al*. [6], and 32 genomes from recent European CC398/ST1232 studies (Denmark, Netherlands, Germany and Hungary) included to facilitate comparison with circulating European clones. All external genomes were retrieved from NCBI GenBank; accession numbers and metadata are provided in Table S5. Main clusters were identified by visual inspection of the cgSNP phylogenetic tree as monophyletic groups exhibiting tight branching patterns indicative of recent divergence.

Raw reads and hybrid genome assemblies were submitted to the NCBI Sequence Read Archive and Genome under BioProject accession number PRJNA1367295.

### Statistics

Statistical analyses were performed to evaluate differences in both continuous and categorical variables. Categorical and continuous variables were compared using chi-square/Fisher’s exact tests and Mann–Whitney U tests, respectively; a *P*-value<0.05 was considered statistically significant. To account for multiple comparisons, *P*-values were adjusted using the Holm–Bonferroni correction method. Ninety-five per cent CIs for proportions were calculated using the Wilson score interval method.

## Results

### Clinical presentation of CC398 in the Czech Republic 2017–2024

Between 2017 and 2024, a total of 59 CC398 isolates from 56 patients were detected with increasing frequency towards the end of the study period (Table 1). Clinical presentation of CC398 isolation was skin and soft tissue infection (SSTI; *n*=31), colonization (*n*=21), respiratory tract infection (*n*=3), urinary tract infection (*n*=2), bone and joint infection (*n*=1) and bloodstream infection (*n*=1). The male-to-female ratio was 37 : 19. The median age of the patient was 40.0 years (interquartile range 20.2 to 56.3 years). The type of hospital unit was ambulance (*n*=30), standard hospitalization (*n*=20), intensive care (*n*=6) and emergency (*n*=3). Healthcare contact was known in 36 cases, and recurrent SSTI was known in 16 cases. Nine patients reported infection of a household/family member, and 14 patients were of foreign origin or had travelled recently outside the country. No contact with livestock was reported, except for one patient reporting contact with horses.

**Table 1.** Trend of CC398 and PVL-positive CC398 among MRSA isolates in the Czech Republic, 2017–2024

	2017*	**2018**	**2019**	**2020**	**2021**	**2022**	**2023**	2024†	All
**All MRSA**	441	151	138	59	59	95	118	68	1129
**CC398 (%, 95% CI)**	11 (2.5, 1.4–4.4)	4 (2.6, 1.0–6.6)	6 (4.3, 2.0–9.2)	0 (0.0, 0.0–6.1)	3 (5.1, 1.7–13.9)	10 (10.5, 5.8–18.3)	13 (11.0, 6.6–17.9)	10 (14.7, 8.2–23.1)	57 (5.0, 3.9–6.5)
**CC398 PVL+ (%, 95% CI)**	1 (0.2, 0.0–1.3)	1 (0.6, 0.1–3.7)	4 (2.9, 1.1–7.2)	0 (0.0, 0.0–6.1)	0 (0.0, 0.0–6.1)	3 (3.2, 1.1–8.9)	6 (5.1, 2.4–10.7)	6 (8.8, 4.1–17.9)	21 (1.9, 1.2–2.8)
**ST1232-MRSA-V (%, 95% CI)**	1 (0.2, 0.0–1.3)	1 (0.6, 0.1–3.7)	4 (2.9, 1.1–7.2)	0 (0.0, 0.0–6.1)	0 (0.0, 0.0–6.1)	4 (4.2, 1.6–10.3)	7 (5.9, 2.9–11.7)	8 (11.8, 6.1–21.5)	25 (2.2, 1.5–3.2)
**ST398-MRSA-IVa (%, 95% CI)**	2 (0.4, 0.1–1.6)	0 (0.0, 0.0–2.5)	0 (0.0, 0.0–2.7)	0 (0.0, 0.0–6.1)	2 (3.4, 0.9–11.5)	3 (3.2, 1.1–8.9)	3 (2.5, 0.9–7.2)	1 (1.5, 0.3–7.9)	11 (1.0, 0.5–1.7)

*For 2017, the proportion of CC398 is derived from Tkadlec *et al. *[14].

†Collected up to the end of June 2024.

Ninety-five per cent CIs (95% CIs) for proportions were calculated using the Wilson score interval method.

### Antimicrobial resistance, resistance and virulence genes

All MRSA isolates carried *mecA*, and 56 isolates carried *blaZ*. Tetracycline resistance (*n*=52) was mediated by *tet*(K), *tet*(M) or both. Gentamicin-resistant isolates (*n*=13) carried *aac*(6′)-*aph*(2″) (*n*=11) or *aph*(2″)-Ia (*n*=2). Resistance to erythromycin/clindamycin (*n*=32) was predominantly associated with the presence of *erm*(A). Isolates resistant to clindamycin only (*n*=15) carried a combination of *lnu*(B) (*n*=15) and *lsa*(E) (*n*=15). Other resistance determinants, including *vga*(E), *dfrG*, *dfrK* and fluoroquinolone-conferring mutations (*gyrA*/*grlA*), were identified across the collection (Table S1). PVL-coding genes (*lukS-PV* and *lukF-PV*) were present in 23 isolates, and the genes of the immune evasion cluster (IEC) were detected in 34 isolates, predominantly in ST1232 and ST398-MRSA-IVa.

### Phages, plasmids and MGEs

All isolates carried one to four phages, most notably phage similar to phi2958PVL (Sa2int) in all PVL-positive ST1232 and P282 (Sa3int) associated with IEC type B. IEC type E in seven ST398-MRSA-IVa isolates was carried by phage SA345ruMSSAST8 (Table S1). Plasmids (rep5a, rep7a, rep16 and repUS43) and various transposons (e.g. Tn554 and Tn6009) were identified, with certain elements showing lineage-specific distribution (Table S1).

### Clonal structure

Isolates were assigned to two sequence types: ST398 (*n*=32), carrying SCC*mec* types IVa (2B) (*n*=11) or Vc (5C2 and 5) (*n*=21), and ST1232 carrying exclusively type V (5C2) (*n*=25), with two ST1232 isolates being methicillin-sensitive. Isolates belonged to the following CC398-related *spa* types by conventional PCR-based *spa* typing: t011 (*n*=18), t034 (*n*=40) and t898 (*n*=1). ST1232 isolates were detected with an increasing frequency towards the end of the study period. Patients with ST1232 were younger and more likely to have SSTIs, recurrent infections, travel history, household contact with others who had MRSA infections or contact with socially disadvantaged groups. ST1232 isolates were significantly more often resistant to erythromycin and clindamycin. ST398 isolates, on the other hand, were more likely to originate from colonization and patients with recent healthcare contact; isolates were more often ofloxacin, gentamicin and tetracycline resistant (Table 2).

**Table 2.** Demographics of patients with ST398 vs ST1232, Czech Republic, 2017–2024. Demographic and antimicrobial resistance characteristics of CC398 MRSA isolates from patients from the Czech Republic stratified by clonal background (ST398 vs ST1232). Data are presented as numbers (%) for categorical variables and as medians (IQR) for continuous variables. *P*-values were calculated using the chi-square or Fisher’s exact test for categorical variables and the Mann–Whitney U test for continuous variables, as appropriate. For multiple comparisons, the Holm–Bonferroni correction of *P*-values was applied. Statistically significant *P*-values are in bold

	ST398 (*n*=32)	ST1232 (*n*=27)	*P*-value (adjusted)	All (*n*=59)
Age (years)				
Median (IQR)	49.9 (28.0–65.2)	31.9 (14.2–42.7)	**0.005 (0.005)**	40.0 (20.2–56.3)
Gender (%)			0.933 (0.933)	
Males	21 (65.6)	18 (66.7)		39 (66.1)
Females	11 (34.4)	9 (33.3)		20 (33.9)
Clinical condition (%)				
Colonization	12 (37.5)	4 (14.8)	0.078 (0.233)	16 (27.1)
SSTI	13 (40.6)	20 (74.1)	**0.010 (0.040)**	33 (55.9)
Recurrent SSTI	3 (9.4)	13 (48.1)	**0.001 (0.006)**	16 (27.1)
RTI	5 (15.6)	1 (3.7)	0.205 (0.409)	6 (10.2)
Other (UTI, BSI, BJI)	2 (6.3)	2 (7.4)	1.000 (1.000)	4 (6.8)
Unit (%)				
Standard	11 (34.4)	9 (33.3)	0.933 (1.000)	20 (33.9)
Ambulance	17 (53.1)	13 (48.1)	0.703 (1.000)	30 (50.8)
IC	4 (12.5)	2 (7.4)	0.678 (1.000)	6 (10.2)
Emergency	0 (0.0)	3 (11.1)	0.09 (0.36)	3 (5.1)
Risk factors (%)				
Healthcare contact	29 (90.6)	7 (25.9)	**<0.001 (<0.001)**	36 (61.0)
Foreigner or travel history	3 (9.4)	11 (40.7)	**0.006 (0.019)**	14 (23.7)
Contact with socially disadvantaged groups	0 (0.0)	6 (22.2)	**0.007 (0.019)**	6 (10.2)
Household infection	2 (6.3)	7 (25.9)	0.066 (0.066)	9 (15.3)
Resistances (%)				
Erythromycin	5 (15.6)	27 (100.0)	**<0.001 (<0.001)**	32 (54.2)
Clindamycin	20 (62.5)	27 (100.0)	**<0.001 (<0.001)**	47 (79.7)
Ofloxacin	11 (34.4)	0 (0.0)	**<0.001 (0.002)**	11 (18.6)
Tetracycline	32 (100.0)	20 (74.1)	**0.003 (0.005)**	52 (88.1)
Gentamicin	11 (34.4)	2 (7.4)	**0.025 (0.025)**	13 (22.0)
Multiplicity of resistance* – median (IQR)	4 (3–4)	4 (3–4)	0.147 (0.147)	4 (3–4)

* Multiplicity of resistance was calculated as the sum of antimicrobial categories (as defined in the definition of *S. aureus* multiresistance [39]) to which the isolate exhibited resistance.

BJI, bone and joint infection; BSI, bloodstream infection; IC, intensive care; IQR, interquartile range; RTI, respiratory tract infection; SSTI, skin and soft tissue infection; UTI, urinary tract infection.

### Whole-genome MLST clonal analysis

**Fig. 1.**
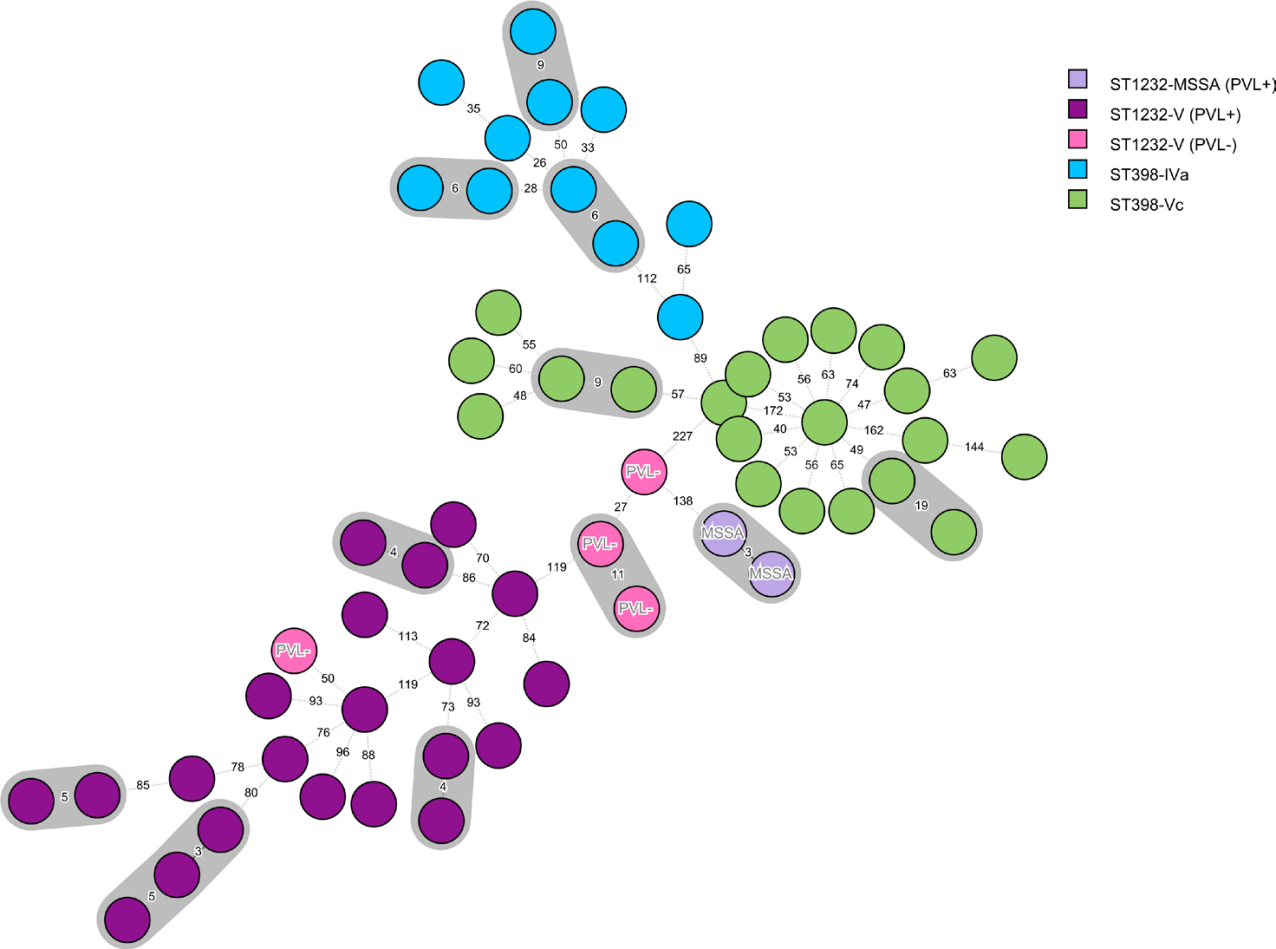
MST based on wgMLST analysis of 59 Czech CC398 isolates. The tree includes 11 isolates from a previous study [14]. MST was constructed using Bionumerics v8.1 (bioMérieux, Applied Maths) using 3,897 loci (isolates' allelic profiles are presented in Table S4). The nodes represent genomes of individual isolates, and branch labels represent allelic distances between neighbouring isolates. The colour of the nodes indicates different genetic backgrounds (STs, SCC*mec* types). PVL-, isolate lacking PVL; MSSA, methicillin-susceptible *S. aureus*.

wgMLST analysis revealed 11 clusters of 2 (*n*=10) or 3 (*n*=1) isolates that differ in less than 24 alleles, indicating recent transmission (Fig. 1). Among the six ST1232 clusters, two were associated with household transmission and the other two with socially disadvantaged groups. One cluster of ST398-Vc consisted of isolates from two patients sharing the same room. Three clusters of ST398-IVa were identified (see below). The 24-allele threshold was applied conservatively based on previous studies. However, a marginal increase of this threshold would result in the expansion of existing clusters by incorporating additional isolates: (i) three PVL-negative ST1232 isolates originating from socially disadvantaged groups would merge into one cluster at ≥27 alleles, and (ii) three and (iii) five ST398-IVa isolates from patients with chronic underlying conditions (oncological disease, cystic fibrosis and renal transplant) would cluster at ≥26 and ≥28 alleles, respectively. For the remaining isolates, the allelic difference to the closest neighbour did not indicate recent transmission (Tables S3 and S4).

### Persistent ST398-IVa carriage

Three clusters of ST398-IVa identified by wgMLST consisted of isolate pairs from the same patient collected in different calendar years and from distinct anatomical sites (respiratory aspirate and urine; sputum and nasal swab; nasal and throat swab, respectively); these were counted as independent non-repetitive cases per the definition provided in the Methods. Time between isolations was 25 (9 allelic differences), 22 (6 allelic differences) and 5 (6 allelic differences) months, respectively, documenting the ability of ST398-MRSA-IVa to colonize humans for an extended period of time. For all three patients, resistance gene content, virulence gene repertoire, plasmid replicon composition and SCC*mec* cassette type remained stable between early and late isolates. Minor discrepancies in phage assignments, presence of ISEnfa4 and adhesin-encoding genes with repetitive domains (*cna*, *clfB*) are attributable to sequence similarity between related phages and the inherent limitations of reference-based detection and short-read assembly rather than genuine genomic change. Clinically, all three patients had chronic underlying conditions (cystic fibrosis, *n*=2; immunosuppression post-transplant, *n*=1) and presented with repeated colonization rather than invasive infection, consistent with long-term asymptomatic carriage under antimicrobial pressure.

### cgSNP-based comparative analysis

**Fig. 2.**
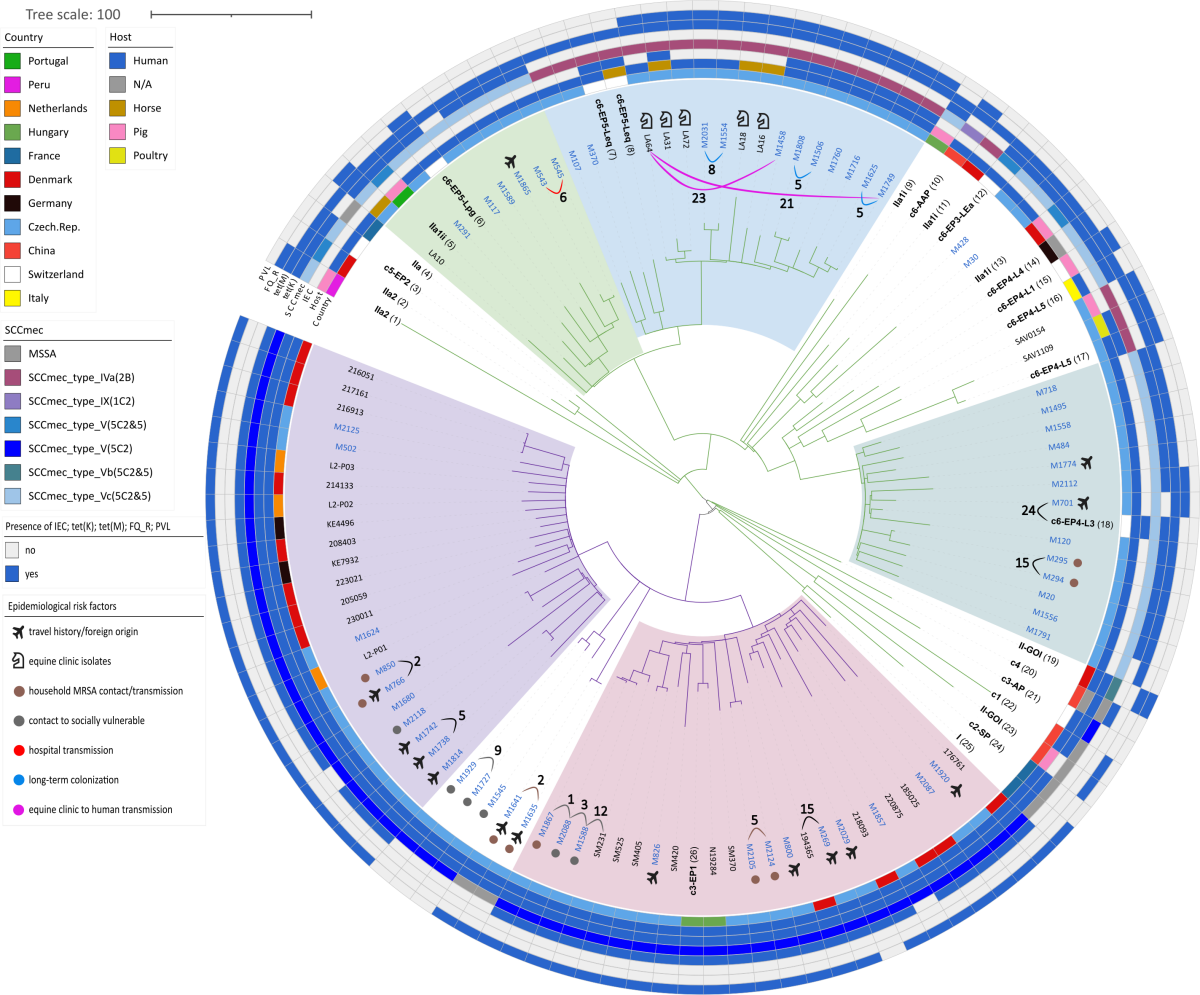
ML tree of 117 CC398 isolates based on cgcgSNP analysis. The tree was constructed based on core-genome alignment (Roary v3.13.0) of annotated genomes (Bakta v1.9.4) after removal of recombinant regions (Gubbins v3.2.1); a maximum likelihood phylogeny was inferred using RAxML (v8.2.12) as implemented in Gubbins, visualized and annotated in iTOL (v7.2.1). The tree includes 59 isolates from this study (bold blue), 32 isolates from recent studies of CC398/ST1232 in Europe and 26 isolates representing phylogenetic lineages of CC398 described previously [5,6]. Isolates representing the CC398 lineages are indicated by the name of the lineages (bold) with a number in brackets referring to Table S5, where the name of the corresponding isolate and accession number can be found. ST398 and ST1232 branches are in green and purple, respectively. Major clusters are indicated by light pink (ST1232_1), light purple (ST1232_2), light green (ST398_1), light blue (ST398_2) and light green/blue (ST398_3) shading. Aeroplane icons denote isolates associated with travel history or foreign patient origin. Horse icons represent cases linked to an equine clinic environment. Coloured circles next to isolate ID indicate specific epidemiological risk factors or patient backgrounds (e.g. socially vulnerable populations and household MRSA contact). Closely related isolates are linked by annotated arcs to highlight transmission chains across the tree topology. The numeric labels on these arcs indicate the genomic distance in SNPs. The colour of the connecting arcs represents the suspected transmission setting. Black arcs denote cases with no known associated risk factor. PVL, Panton–Valentine leucocidin; FQ_R, resistance to fluoroquinolones (determined by antimicrobial susceptibility testing or by the presence of genetic markers); IEC, immune evasion cluster.

Further phylogenetic reconstruction based on the core genome alignment includes an additional 58 isolates selected from other studies (Table S5). ML tree shows splits of CC398 isolates into five main clusters (Fig. 2; for the distance matrix, see Table S6). ST398 lineage exhibited greater diversity, with isolates from human patients interspersed among equine and porcine reference strains. A prominent sublineage (ST398_2) was shared between human patients and equine clinics, with some human patient–equine clinic pairs differing by as few as 21–23 SNPs (e.g. LA64/M1749 and LA64/M1458). The high degree of genetic similarity suggests recent cross-species transmission. Notably, approximately half of the isolates in this sublineage harboured the IEC, suggesting a human adaptation. In contrast, ST398 isolates from other sublineages clustered with classical swine-associated LA-MRSA reference genomes; these were characterized by the presence of SCC*mec* Vc and *tet*(M) and a near-complete absence of the IEC.

The ST1232 lineage formed two main clusters (ST1232_1 and ST1232_2), within which Czech isolates were interspersed among those from diverse international origins. Rather than a single expansion, the phylogenetic distribution suggests multiple independent introduction events, often linked to travel history. This is further supported by our clinical data, where several patients reported recent travel or were of foreign origin, primarily from South-East Asia. Notably, we identified several distinct micro-clusters (0–5 SNPs) indicating active local transmission within households and among patients with links to vulnerable populations.

### Complete genome analysis of representative isolates

**Fig. 3.**
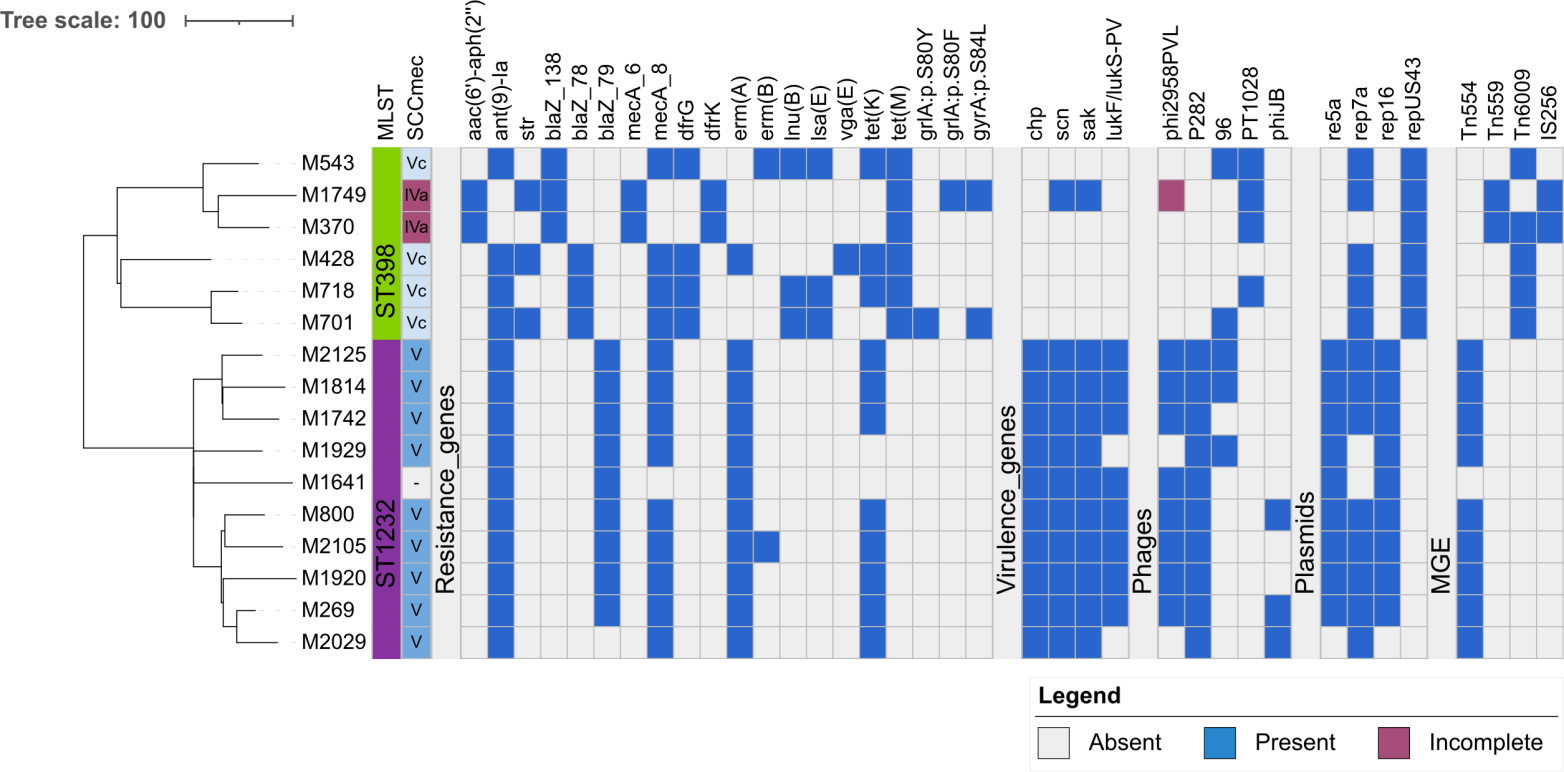
ML tree of cgcgSNP analysis and genetic characteristics of 16 Czech CC398 isolates representing CC398 phylogeny. Hybrid assemblies of complete genomes were used. The tree was constructed based on core-genome alignment (Roary v3.13.0) of annotated genomes (Bakta v1.9.4) after removal of recombinant regions (Gubbins v3.2.1); a maximum likelihood phylogeny was inferred using RAxML (v8.2.12) as implemented in Gubbins, visualized and annotated in iTOL (v7.2.1). The presence of resistance (ResFinder v4.7.2) and virulence (VirulenceFinder v2.0) genes, phages (PHASTEST), plasmids (PlasmidFinder v2.1) and other MGEs (MobileElementFinder v1.0.3), as well as sequence types (MLST v2.0) and SCC*mec* types (SCCmecFinder v1.2), is indicated.

Sixteen isolates representing the major phylogenetic clusters of CC398 were subjected to both short- and long-read sequencing. The resulting hybrid assemblies were used to analyse genome organization, presence of MGEs and their content. ML tree of hybrid assemblies was used to vizualize the main differences between CC398 subgroups in our study (Fig. 3; for distance matrix, see Table S7). Plasmids were present in all but two isolates. A plasmid containing rep5a and rep16 of 20 kb size carrying *blaZ* was present in eight hybrid assemblies of ST1232. In one ST1232, similar plasmid of 35 kb containing additional resistance genes [*erm*(B); *dfrE*; *aph*(2″)-Ia] was present. In the remaining ST1232 isolate, the plasmid was not detected. Among ST398, a smaller plasmid of 4.5 kb carrying the *str* gene was found in three isolates.

## Discussion

While CC398 MRSA was first reported in Central Europe two decades ago [32], it has remained largely associated with livestock. Our 7-year surveillance documents a significant epidemiological shift in the Czech Republic, characterized by the emergence of human-adapted, PVL-positive ST1232 frequently isolated from younger patients with SSTI, recent travel history or contact with socially disadvantaged groups. In addition, we observed the emergence of ST398 sublineages, frequently isolated from older patients with healthcare contact or chronic conditions. This shift from a CC398 population being preferentially represented by livestock-associated sublineages to a multifaceted population consisting of concurrent human- and livestock-associated sublineages mirrors recent reports from Northern and Western Europe [9,10] but highlights a more rapid clinical expansion than previously recognized.

Previous phylogenetic analyses of CC398 have revealed the existence of multiple evolving human- and animal-adapted sublineages [1,5, 6]. Despite considerable genetic diversity, CC398 MRSA has for a long time remained predominantly associated with livestock in Europe [33]. In our study, ST398 isolates exhibited a dual epidemiology. One portion resembled classical LA-MRSA [PVL-/IEC-negative, *tet*(M)-positive], likely reflecting sporadic zoonotic spillover [33]. However, a distinct subgroup carrying SCC*mec* IVa and IEC was identified. This lineage (C6-EP5-Leq) has been linked to equine reservoirs [5], and our genomic analysis confirmed its circulation in Czech equine clinics [34]. Notably, a significant proportion of these isolates harboured the IEC type E, indicating adaptation to the human host. Moreover, this lineage was identified in patients with chronic conditions (e.g. cystic fibrosis and transplant history), often presenting as long-term colonization lasting up to 25 months. These isolates frequently exhibited fluoroquinolone resistance, a trait that has been identified as a major driver for the evolutionary success of various healthcare-associated MRSA lineages under the selective pressure of clinical environments [35]. Our findings suggest that environments with high antimicrobial pressure and close contact, whether human hospital or equine clinics, may serve as critical niches for the persistence of CC398 and facilitate its cross-species transmission.

The expansion of ST1232-MRSA-V is particularly concerning. Unlike previous sporadic reports from European countries [11,13], our longitudinal data document a steady increase in this highly virulent clone, primarily causing recurrent SSTIs. Despite the smaller sample sizes in certain years, the lack of overlap between the 95% CIs of the initial and final years of the study confirms that the increase in ST1232 prevalence represents a significant shift rather than a random fluctuation. While early European cases were predominantly linked to South-East Asia [9,10], less than half of our cohort fit this pattern. This indicates that following multiple introduction events, ST1232 has become established within the local population.

The identification of household clusters and cases among vulnerable groups (e.g. individuals experiencing homelessness or intravenous drug use) mirrors the early transmission dynamics of successful CA-MRSA clones like USA300 [36,37]. This trend may be further exacerbated by current socio-economic shifts in Europe, where 21% of the population still remains at risk of poverty or social exclusion [38]. Economic instability, coupled with significant migration flows following the conflict in Ukraine, has expanded marginalized populations living in high-density environments. Such conditions are known to facilitate the rapid dissemination of CA-MRSA, potentially replicating the epidemiological trajectory of the USA300 clone [36]. Our findings suggest that ST1232 may possess a similar potential for a major community-associated epidemic in Europe, shifting the status of CC398 from a sporadic zoonotic threat to a sustained public health challenge.

This study has several limitations. With the exception of the 2017 multi-centre survey [14], data were collected from a single tertiary referral centre (Motol University Hospital, Prague). Although this centre serves a large and diverse patient population, the findings may not be fully representative of the national epidemiological landscape, and referral bias towards more complex cases cannot be excluded. In particular, the apparent increase in CC398 prevalence towards the end of the study period should be interpreted with this in mind. Multi-centre prospective surveillance would be required to confirm the national trends suggested by our data.

## Conclusion

These findings underscore the evolving 'One Health' threat of CC398, where livestock and human community networks serve as overlapping reservoirs for increasingly virulent MRSA sublineages.

## Supplementary material

10.1099/mgen.0.001741 Supplementary Material 1.

## Abbreviations

CA: community-associated
CC398: clonal complex 398
cgSNP: core-genome SNP
IEC: immune evasion cluster
iTOL: Interactive Tree of Life
LA-MRSA: livestock-associated methicillin-resistant *Staphylococcus aureus*
MGEs: mobile genetic elements
MST: minimum spanning tree
RTI: respiratory tract infection
SSTI: skin and soft tissue infection
